# A technique avoiding cardioplegia delivery complications: a case using systemic hyperkalemia cardiopulmonary bypass combined with circulatory arrest

**DOI:** 10.1051/ject/2024027

**Published:** 2024-12-20

**Authors:** Tomohisa Takeichi, Yoshihisa Morimoto, Akitoshi Yamada, Takanori Tanaka

**Affiliations:** 1 Department of Clinical Engineering, Kitaharima Medical Center 926-250, Ichiba-cho Ono-shi Hyogo 675-1392 Japan; 2 Department of Cardiovascular Surgery, Kitaharima Medical Center 926-250, Ichiba-cho Ono-shi Hyogo 675-1392 Japan

**Keywords:** Cardiopulmonary bypass (CPB), Systemic hyperkalemia, Circulatory arrest, Cardioplegia delivery complications

## Abstract

We conducted a high-risk redo mitral valve replacement through a right mini-thoracotomy without rib spreading (redo-MICS MVR) under systemic hyperkalemia combined with circulatory arrest to circumvent complications associated with cardioplegia delivery. The patient, a 75-year-old man, had a predicted mortality rate of 20%. Initial antegrade cardioplegia successfully induced cardiac arrest, which was administered every 30 min. However, upon infusion of the second dose of cardioplegia, the aortic root pressure was approximately 20 mmHg. Despite multiple attempts to re-cross the clamp, the aortic root pressure did not improve. Consequently, retrograde cardioplegia was considered, but due to significant adhesion of the inferior vena cava, this approach was abandoned. Thus, the procedure was altered to utilize systemic hyperkalemia without aortic cross-clamping (ACC). Given the preoperative transesophageal echocardiography (TEE) diagnosis of mild aortic regurgitation, maintaining a clear surgical field was challenging, necessitating the combination of redo-MVR with circulatory arrest. This case exemplifies the successful management of cardioplegia delivery complications using systemic hyperkalemia and circulatory arrest, resulting in a favorable postoperative recovery for the patient.

## Introduction

A meta-analysis demonstrates that minimally invasive cardiac surgery (MICS) for redo cases is associated with a reduced incidence of in-hospital mortality, reintervention for bleeding, and acute renal failure compared to median sternotomy for redo mitral valve surgery [1]. At our institution, we actively employ totally endoscopic MICS for redo cases, aiming to mitigate risks and optimize patient outcomes. However, it is crucial to exercise caution regarding aortic cross-clamping (ACC) due to the adhesions frequently present around the aorta in redo cases. Complications in cardioplegia delivery can be fatal. In such scenarios, beating heart (BH) surgery or ventricular fibrillation (VF) is often performed. A concern with utilizing VF is subendocardial hypoperfusion. Blood flow to the subendocardium occurs during diastole, and the compressive force exerted on the subendocardial muscle by fibrillation restricts blood flow and oxygen delivery to the myocardium during the diastolic phase [2]. Consequently, myocardial edema increases in the static diastolic state, potentially leading to cardiac dysfunction. At our institution, we employ systemic hyperkalemia cardiopulmonary bypass (CPB) [3]. In this case, to avoid complications in cardioplegia delivery due to incomplete ACC, we performed mitral valve replacement (MVR) under systemic hyperkalemia CPB. Circulatory arrest was also incorporated to address poor surgical visualization resulting from aortic regurgitation (AR).

This study was approved by the Institutional Review Board at Kitaharima Medical Center (IRB-0562) with the waiver of informed consent.

## Case report

**Figure 1:**
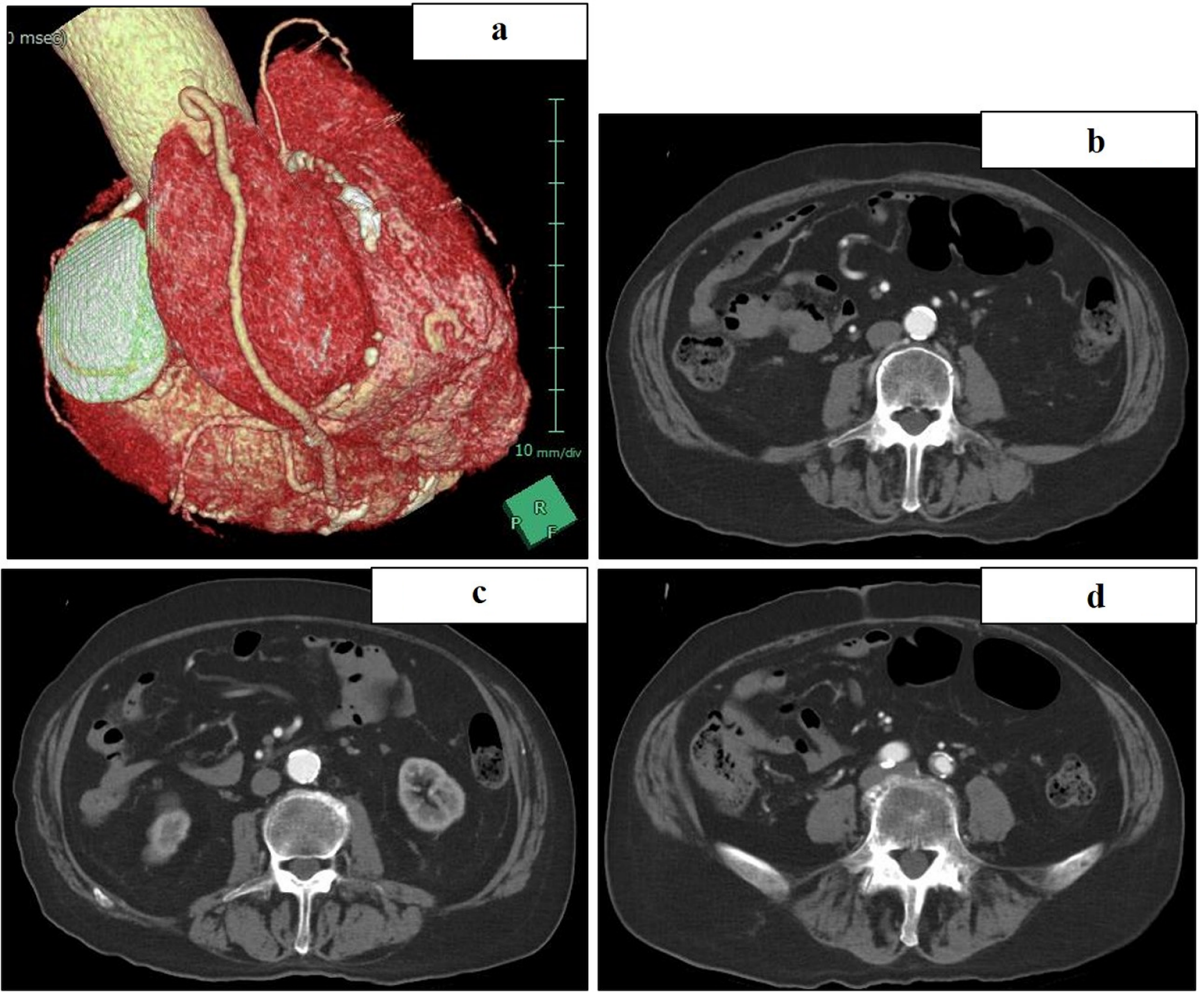
(a) Preoperative 3D CT images. (b–d), preoperative contrast-enhanced CT images from the abdominal aorta extending to the iliac arteries. CT: Computed Tomography.

A 75-year-old male patient (height: 166.4 cm; weight: 66.2 kg), who had previously undergone percutaneous coronary intervention (PCI) to the left anterior descending artery (LAD) and coronary artery bypass grafting (CABG) with a saphenous vein graft to the posterior descending branch (SVG-4PD) combined with mitral valve replacement (MVR) 12 years prior, was diagnosed with severe mitral regurgitation and mild aortic regurgitation via TEE. Three-dimensional computed tomography (CT) demonstrated the SVG traversing the right atrium, raising concerns about the feasibility of retrograde cardioplegia (Figure 1a). Additionally, contrast-enhanced CT scans revealed calcification extending from the abdominal aorta to the iliac arteries (Figures 1b–1d). The patient exhibited a low left ventricular ejection fraction (30%), chronic kidney disease (estimated glomerular filtration rate: 21 mL/min/1.73m^2^; creatinine: 2.42 mg/dL), and a predicted mortality rate of 20%. The surgical strategy entailed a totally endoscopic redo-MVR.

**Figure 2:**
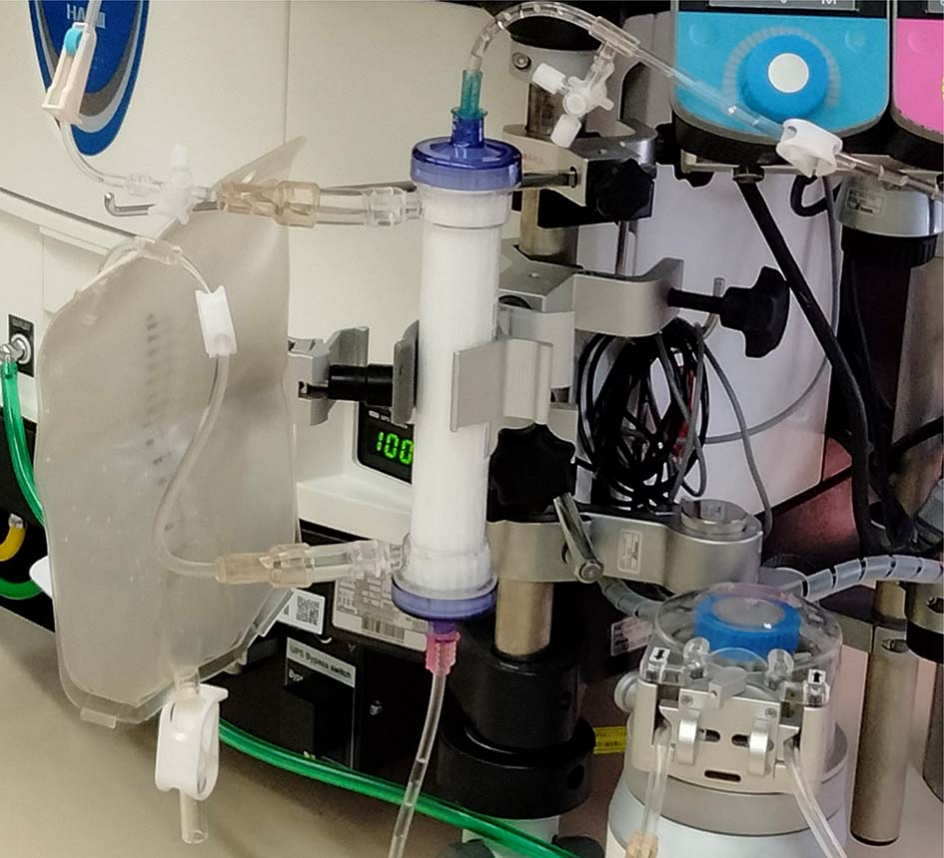
Gravity drainage hemodiafiltration method.

Upon induction of general anesthesia, the patient underwent redo-MVR via a right mini-thoracotomy without rib spreading. CPB was initiated with a 23/25Fr venous cannula (MICS Cannulae; LivaNova, Tokyo, Japan) in the right femoral vein and an 18Fr arterial cannula (PCKC-A, MERA, Tokyo, Japan) in the right femoral artery. The pump flow rate was maintained at 2.0 L/min/m^2^, with mean arterial pressure stabilized at 70 mmHg. To mitigate postoperative oliguria due to impaired renal function, continuous administration of furosemide (45mL of 20% mannitol + 50 mg of furosemide) at 10 mL/h was planned alongside intraoperative hemodialysis via gravity drainage hemodiafiltration (GHDF) using a saline solution as dialysate (Figure 2). Although the target temperature was initially set at 30.0 °C, it was adjusted to 28.0 °C due to anticipated difficulties with ACC and the patient’s mild aortic regurgitation. Cold blood cardioplegia was administered via the aortic root after cross-clamping the ascending aorta, resulting in prompt cardiac arrest.

The left atrium was opened, the artificial valve was removed, and antegrade cardioplegia was re-administered after repositioning the retractor holding the left atrium. However, aortic root pressure remained low at 20 mmHg despite multiple attempts at re-cross-clamping. Efforts to switch to retrograde cardioplegia were thwarted by significant adhesions of the inferior vena cava (IVC) and the bypass graft (SVG-4PD), rendering the procedure too challenging. Consequently, systemic hyperkalemia without ACC was employed. To ensure adequate cardioplegia delivery, thorough adhesiolysis was imperative to avoid damaging the SVG, with direct retrograde cardioplegia potentially being insufficient.

**Figure 3:**
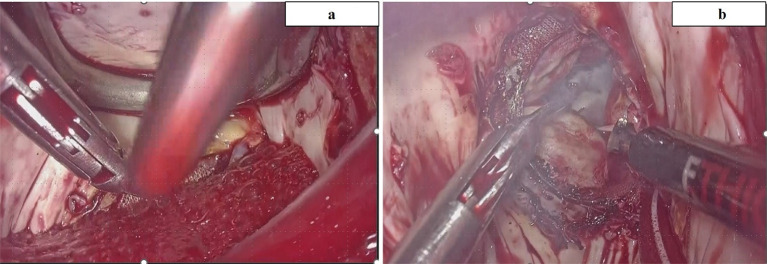
(a) Showing did not get a good vision because of aortic regurgitation after de-clamping ACC. (b) After circulatory arrest combined with systemic hyperkalemia without ACC we could get a good view. ACC: Aortic cross-clamping.

After de-clamping, a mixed solution (500 mL of bicarbonate ringer solution with 50 mL of KCL 10 mEq/L, 20 mL of MgSO_4_, and 100 mg of 2% lidocaine) was continuously infused from CPB to maintain cardiac arrest, targeting a blood potassium level of 9.0 mEq/L. Due to the influence of aortic regurgitation, maintaining a clear surgical field was challenging; hence, MVR was performed in conjunction with circulatory arrest (Figures 3a and 3b). Bladder and eardrum temperatures were recorded at 26.0 °C and 23.0 °C, respectively. MVR was executed with intermittent circulatory arrest periods of 3–5 min during annulus suturing and prosthetic valve sizing, totaling 39 min of circulatory arrest. Upon confirming a vent through the mitral valve in the left ventricle, continuous mixed solution infusion ceased, maintaining cardiac arrest until the left atrium was closed. In the event of heartbeats, 100 mg of 2% lidocaine was infused to re-induce cardiac arrest by inhibiting voltage-dependent Na^+^ channels.

Following the closure of the left atrium, rewarming commenced alongside potassium level reduction and GHDF initiation. At that juncture, potassium concentration was 8.5 mEq/L. The combination of continuous furosemide and GHDF effectively reduced potassium levels instantaneously. CPB was weaned using 3.1γ of dobutamine and 0.03γ of noradrenaline. The rewarming duration was 40 minutes, and the time to normalize potassium levels to 5.0 mEq/L was 30 min, using 14000 mL of saline solution in the GHDF dialysate. Cardiac arrest duration was 189 min, and CPB time was 325 minutes, with a post-CPB potassium level of 4.8 mEq/L and a peak level of 10.5 mEq/L. Urine output during CPB was 6.5 mL/kg/h. The patient’s postoperative peak creatine-kinase MB (CK-MB) level was 24.0 ng/dL, and the peak creatinine value was 2.83 mg/dL. Mechanical ventilation duration was 15 h, and ICU stay was 8 days. No major complications, including acute kidney injury, were noted. The postoperative course was uneventful, and the patient was discharged.

Informed consent to report patient information and images was obtained.

## Discussion

Reoperative valve surgery is recognized for its complexity, increased morbidity, and elevated mortality rates, with the challenges in cardioplegia administration significantly affecting patient outcomes [4, 5]. In this case of incomplete ACC, we safely executed a high-risk redo MICS MVR under systemic hyperkalemia combined with circulatory arrest, bypassing the need for ACC. Our prior experience with systemic hyperkalemia during CPB enabled us to promptly address the incomplete ACC scenario utilizing systemic hyperkalemia without ACC [3].

When faced with severe adhesion of the IVC and SVG traversing the right atrium, retrograde cardioplegia poses significant difficulty. In cases where cross-clamping is challenging, techniques such as the BH method or VF arrest can be safely employed [2–4, 6]. However, some researchers have demonstrated that the VF approach is inferior to the BH technique due to its reduced oxygen delivery to the sub-endocardium, leading to suboptimal myocardial protection. Sub-endocardial perfusion occurs during diastole, and the compressive forces of fibrillation impede myocardial blood flow and oxygen delivery during the diastolic phase of VF [2, 7]. Hiraoka et al. reported that intraoperative myocardial protection under mild hypothermia and VF is not inferior to protection achieved with cardioplegic arrest [8].

These strategies are effective provided that aortic insufficiency is no more than mild to moderate. Severe aortic insufficiency complicates the maintenance of a bloodless surgical field and can lead to coronary malperfusion [7, 9]. In this case, mild aortic regurgitation was identified via preoperative TEE. Due to the regurgitation, achieving a clear surgical field without circulatory arrest was unfeasible. In such scenarios, combining circulatory arrest with systemic hyperkalemia CPB can be beneficial, as it ensures uniform myocardial protection compared to fibrillatory arrest. Postoperative creatine kinase-MB (CK-MB) levels were 24 ng/dL, and there are no previous reports of systemic hyperkalemia combined with circulatory arrest without ACC.

Opinions on this approach may vary. In high-risk cases, some surgeons might prefer median sternotomy over MICS to avoid cardioplegia-related complications. However, we posit that MICS for redo cases is safer than median sternotomy. Opting for open conversion to ensure adequate cardioplegia dosing carries the risk of SVG damage, which could be catastrophic. Recent meta-analyses have highlighted the advantages of MICS over median sternotomy for redo cases, deeming it effective even for high-risk patients [1].

Therefore, we believe that our MICS approach to circumvent open conversion was optimal for this patient. Previously, we reported a complex case managed under systemic hyperkalemia [3]. In this instance, despite the addition of circulatory arrest, no major complications were observed, and the postoperative course was uneventful, leading to the patient’s discharge.

We advocate for the utility of this strategy in managing cardioplegia-related issues in complex cases. Nonetheless, there is limited literature on right thoracotomy under systemic hyperkalemia CPB combined with circulatory arrest, indicating the need for further investigation into this technique.

## Data Availability

All available data are incorporated into the article.
